# Providing real-time resources in support of LGBTQ+ and HIV+ populations as information experts on the ECHO hub team: a case report

**DOI:** 10.5195/jmla.2021.1262

**Published:** 2021-10-01

**Authors:** Laura Menard, Chelsea Misquith

**Affiliations:** 1 lmenard@iu.edu, Assistant Director for Medical Education and Access Services, Ruth Lilly Medical Library, Indiana University, Indianapolis, IN; 2 chelsea_misquith@brown.edu, Brown University, Providence, RI

**Keywords:** community health services, telemedicine, sexual and gender minorities, information literacy

## Abstract

**Background:**

Project ECHO (Extension for Community Healthcare Outcomes) is a telehealth initiative that aims to reduce disparities in delivery of health care by leveraging technology and local expertise to provide guidance on specialized subjects to health care providers across the world. In 2018, a new ECHO hub convened in Indianapolis with a focus on health care for individuals in the lesbian, gay, bisexual, trans, and queer (LGBTQ+) populations. This ECHO iteration was one of the first of its kind and would soon be followed by a new human immunodeficiency virus (HIV) ECHO as well.

**Case Presentation:**

In a novel approach, information professionals participated in the early planning stages of the formation of these ECHO teams, which enabled the provision of real-time medical evidence and resources at the point-of-need once the teams were launched. This case study demonstrates proof of concept for including health sciences librarians and/or information professionals in the ECHO as hub team members. In this case study, the authors describe and quantify the value added to the HIV and LGBTQ+ ECHO sessions by the medical librarians, as well as provide a template for how other telehealth initiatives can collaborate with their local health information professionals.

**Conclusions:**

Librarian involvement in Project ECHO over the past three years has been enthusiastically received. The librarians have contributed hundreds of resources to ECHO participants, helped build and curate resource repositories, and expanded the embedded librarian program to an additional two ECHO iterations. ECHO hub team members report high rates of satisfaction with the performance of embedded librarians and appreciate the provision of point-of-need evidence to ECHO participants.

## BACKGROUND

For many decades, health sciences librarians have provided clinical support to health care professionals. Direct contributions of clinical librarians have included assisting health care practitioners in choice of intervention, diagnosis, cost savings, and risk management, among others [1]. Moreover, librarians and information professionals have provided services to support patient care by engaging in searching for literature at the point-of-care, contributing to shared decision-making, and providing access to patient education materials [2]. However, the scholarship shows mixed findings about the effectiveness of librarians' involvement on patient outcomes, and more research is necessary to prove impact [3, 4, 5].

There is, however, ample precedent for librarians working to support clinicians who are engaged in clinical work related to LGBTQ+ and HIV-positive populations. In the past few years alone, health sciences librarians have been involved in many aspects of LGBTQ+ information creation, dissemination, preservation, and delivery. Health sciences librarians have created LGBTQ+-specific health archives, worked to advance LGBTQ+ Wikipedia engagement, built inclusive collections at their institutions, researched better ways to improve access to resources, and created safe spaces at their libraries for LGBTQ+ patrons [6, 7, 8]. The US National Library of Medicine (NLM) has developed multiple, freely available web databases and online resources for health practitioners and the general public to access information about HIV and acquired immunodeficiency syndrome (AIDS) [9]. One such resource is AIDSinfo, which provides access to federally approved practice guidelines, HIV treatment and prevention, patient resources, and community-based organization information [10].

Project ECHO (Extension for Community Healthcare Outcomes) leverages technology to reduce health care knowledge disparities. Launched originally at the University of New Mexico, the ECHO program uses case-based and didactic learning to provide virtual training on health care topics to clinicians all over the US and on a global scale [11]. Librarians at the Ruth Lilly Medical Library in Indianapolis have recently begun to contribute to a new project aimed at reducing health care disparities and providing point-of-need support to practitioners working with diverse and underserved populations.

Meanwhile, with funding from the NLM, a group of librarians at the University of Florida Health Sciences Libraries have partnered with community groups and university affiliates to develop a curriculum for health and social service providers on finding relevant literature about HIV and AIDS [12]. Because these resources are free for other librarians to modify and reuse as learning tools within their institutions and communities, the librarian involved with the HIV ECHO has been taking advantage of these resources to better prepare ECHO participants to care for their patients.

ECHO programs focusing on many different topics have been in existence in some form since 2003, and quite a few attempts have been made in the literature to quantify their impact. According to a systematic review published in 2019, there is some evidence suggesting the success of ECHO as a clinical intervention on patient outcomes relating to hepatitis C, chronic pain, dementia, and type 2 diabetes [13]. However, much of the literature relies on self-reported outcomes and surveys, which tend to suffer from low response rates. This suggests that there are significant opportunities for practitioners and leaders involved in the ECHO project to assess both provider- and patient-related outcomes of ECHO and similar telehealth interventions by developing better mechanisms for quantifying impact and incentivizing feedback. These gaps in the research regarding ECHO and other telehealth initiatives may be of interest to librarians and health sciences informationists who wish to contribute to the body of knowledge through involvement with interdisciplinary research efforts.

## CASE PRESENTATION

At the Indiana University Purdue University Indianapolis (IUPUI), there are several ECHO iterations focusing on different health outcomes and populations currently running. This case presentation will focus on the two that have been in existence for at least a year: LGBTQ+ and HIV. Each ECHO adheres to the “hub and spokes” model, wherein experts at the Indianapolis hub direct the sessions and guide conversations, adding their expertise as necessary. The participants, or “spokes,” are practitioners who may be isolated or practicing in a specialty that requires them to independently participate in continuing education in order to hone their skills. Additionally, participants are encouraged to bring cases to ECHO sessions for discussions and advice on patient care. All participants in IUPUI's ECHO sessions can receive continuing education (CE) credit for attendance and participation.

Embedded librarians were included in hub team meetings from the planning stage forward. Both the HIV and LGBTQ+ ECHO coordinators reached out to request an embedded librarian at least a month prior to the start of the ECHO and included the librarians in each planning session. The LGBTQ+ ECHO has been running since September 2018, with plans to continue as long as funding is available. The HIV ECHO began in February 2019 and is currently in its second year. Each of these ECHO programs has 1.5-hour tele-clinic sessions that take place twice a month. Hub teams for ECHO programs at IUPUI are interdisciplinary and comprised of physicians, social workers, pharmacists, nurses, and other health care practitioners.

**Figure 1:**
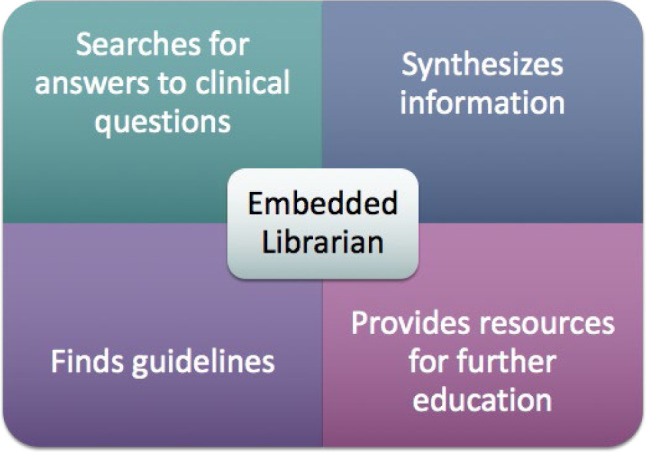
Embedded librarian role in ECHO

The embedded librarians' responsibilities center around providing real-time informational support to the hub team and ECHO participants during ECHO sessions (Figure 1). This duty is mainly relevant during the case presentations and discussions. During these discussions, the librarians search for answers to clinical questions as they arise, synthesize the evidence found, interpret and share information from relevant clinical resources, and ensure that the team has the most up-to-date and high-level evidence to inform patient care. The librarians may also search for and share guidelines (screening, dosage, etc.) and coach practitioners on finding and using this type of resource independently. All resources are uploaded and maintained in individual ECHO Canvas sites.

One surprising request from both hub team members and ECHO participants has been for nonclinical resources to aid in understanding patient populations with whom practitioners may need guidance to provide culturally competent care. ECHO participants have requested educational materials on definitions of gender identities and sexual orientations, resources for unhoused patients, information on legal issues, and nonclinical community support for patients and their families. For these types of questions, the librarians have used resources that are traditionally nonclinical, but which fit the information needs of the session attendees. Some examples of nonclinical resources include books, blogs, zines, housing and legal resources, as well as contact information for local support groups. These resources have been added to a Frequently Asked Questions page on the Canvas sites for participants.

The librarians need to be cognizant of copyright and information sharing restrictions because most ECHO participants are not affiliated with academic institutions. The ECHO hub team initially used Box to share resources with participants. Later, the team switched to Canvas for practical and cost-related reasons. For each session, the librarian reviews any resources referenced during the didactic session or during the discussion for copyright concerns. If materials are freely available to the public or open access, they are added to the Canvas site for attendees to use. If materials are available only through a subscription model or are subject to copyright restrictions, a citation is added to the site and attendees are encouraged to use interlibrary loan at their own institutions to access a copy of the resource.

**Figure 2:**
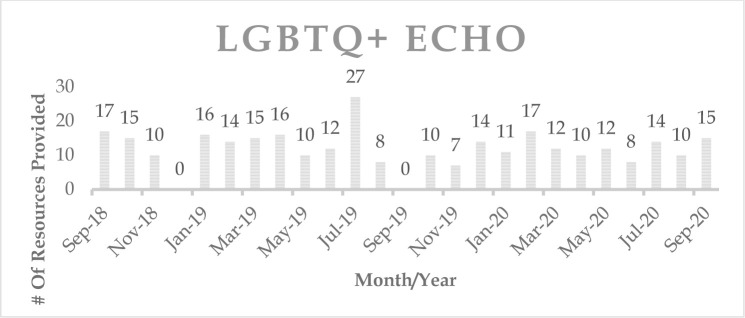
Number of resources provided per month in LGBTQ+ ECHO

Over the course of two years, the embedded librarian for the LGBTQ+ ECHO provided 300 resources (articles, websites, guidelines, books, presentations, etc.) during 48 sessions to the ECHO participants (Figure 2). In December of 2018 and September of 2019, no LGBTQ+ ECHO sessions took place, but every other month's resource numbers are indicative of resources provided for two sessions.

**Figure 3:**
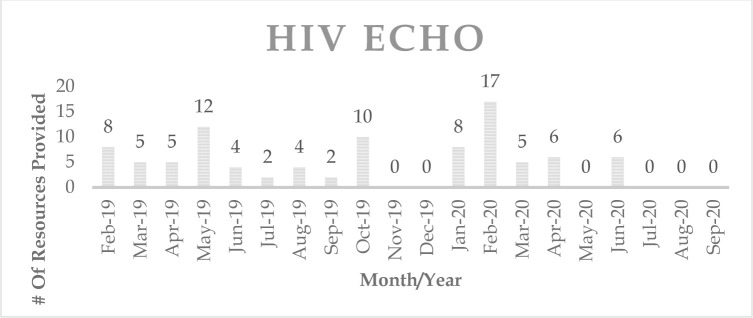
Number of resources provided per month in HIV ECHO

The embedded librarian for the HIV ECHO provided 94 resources (studies, guidelines, recommendations, websites, drug interaction checkers) over 14 monthly sessions to the HIV ECHO participants (Figure 3). Every month's resource numbers are indicative of resources provided for one session.

According to an informal request for feedback disseminated to members of the hub team in 2019, librarian involvement in the LGBTQ+ ECHO has been impactful and beneficial. Every member of the hub team responded to the request for feedback (n=13). When asked to agree or disagree with the statement “As a member of the hub team, the medical librarian adds value to ECHO sessions,” twelve out of the thirteen total hub team members either agreed or strongly agreed. In a free text response, nine out of the thirteen respondents cited real-time informational support as a reason that they would recommend librarian involvement to other ECHO hub teams. Other responses to this question mentioned that librarians can help support evidence-based practice as well as preserve and disseminate the resources referenced and utilized during the session for participants' future use. See Appendix A for the full text of the informal survey.

## DISCUSSION

Both the LGBTQ+ and the HIV ECHO programs are in their second year at IUPUI in the fall of 2020. The librarians on the LGBTQ+ and HIV ECHOs, in addition to providing resources for hub team members and participants, have become more involved with the clinicians as they have become more aware of the scope of a medical librarian's skills throughout the duration of the ECHO. The librarians have performed several literature searches to support the hub team as they prepare didactic sessions for presentation and research for publication.

In addition, both librarians have contributed to their respective ECHOs as subject experts. The librarian for the LGBTQ+ ECHO presented her own didactic session at the beginning of 2020, focusing on freely available resources for patients and providers related to LGBTQ+ health outcomes. This presentation was subsequently uploaded into the institutional repository to facilitate access [14]. The librarian on the HIV ECHO gave a short presentation at the first session of year 2, highlighting information resources that healthcare providers could use to search for relevant literature, guidelines, and fact sheets for themselves and for patient education.

Since the librarian first began work with the LGBTQ+ ECHO in 2018, and because of her support and demonstration of the skillset and resources she could provide, the ECHO program at IUPUI has requested librarians to participate in three additional ECHO iterations: HIV, cancer, and a COVID-19 rapid response [15]. This expansion in scope of librarian involvement is proof of concept that the integration of a librarian into the expert hub team is beneficial to ECHO organizers and participants. Moreover, while the results of this program's informal surveys are not conclusive, they may suggest that librarian participation in ECHO or similar initiatives may be a good avenue for health sciences librarians to interact with practitioners and demonstrate information literacy skillsets with which these individuals may not otherwise have become familiar.

This collaboration has not been without its challenges. Given varying institutional access restriction, choosing a repository for resources that would be readily accessible to all was difficult. For example, while Google Drive offers free storage in an interface that is familiar to many healthcare practitioners, Google web applications are blocked at many hospitals and therefore would not fit our purposes. Because Indiana University (IU) had an institutional subscription to Box, the team initially organized materials there for distribution. However, because not all practitioners had an existing Box account, materials stored in this space were accessed less as time went by. Finally, in 2021, the ECHO iterations at IU made the decision to transfer content and instructions on claiming CE credit to Canvas. This helped us to organize resources easily by topic and date, as well as gave practitioners added incentive to create an account and engage with the Canvas course.

The second challenge was providing access to resources. For each of the ECHO iterations, the embedded librarian needed to be well versed in the limits of fair use and resource dissemination. The most significant challenge in this area was the limitations on sharing articles in Canvas or Box to a group of participants with varying levels of institutional access to journals and databases. When available, librarians endeavored to find open access articles to share. However, there were times when the best evidence was paywalled and accessible only to those with a university affiliation. In these cases, the librarian summarized the evidence for the participants and provided a clear citation that could be sent to a participant's interlibrary loan service if they wished to read the full text of the article. Due to the fast-paced nature of clinical care, however, many practitioners do not have the time to undergo such a rigorous process to access resources relevant to patient care [16]. This case study demonstrates a real, high-stakes example of the importance of open access publications to health sciences librarians providing resources at the point of need.

In the future, librarians and information professionals should look to ECHO hub teams at their own institutions to explore the possibility of partnering to provide relevant information during and around the sessions. Practically speaking, librarians should evaluate their ability to commit two to four hours each month to attending these sessions either in person or virtually, as well as roughly an hour outside of sessions to summarize and present answers to questions that arose during the session. As Project ECHO and other telehealth initiatives continue to expand in the wake of the COVID-19 pandemic, the demand for information professionals to take on the roles outlined in this case report will likely grow [17]. While this is an opportunity to expand services for academic and clinical librarians, it is important to be cognizant of the scalability of the services provided. For example, a librarian may wish to limit in-person session attendance to once a week, month, or quarter, dependent on the other demands of their position. As a member of the ECHO hub team, the librarian should feel comfortable sharing their skillsets as well as advocating for the value of their time.

As Project ECHO moves into its third year at IUPUI, the Ruth Lilly Medical Library ECHO team has plans to evaluate the impact of the embedded librarian model beyond this case study. In the next year, the four embedded librarians now working with project ECHO will develop, refine, and deploy a survey for both hub team members and ECHO participants aimed at ascertaining the attitudes of the ECHO participants toward the embedded librarian model. The team will also include questions evaluating participants' use of the resources provided in ECHO sessions. By continuing to survey participants, the librarians hope to gain a better understanding of the information needs of practitioners who participate in this and similar telehealth initiatives.

Project ECHO provides an opportunity for health sciences librarians to impact patient care, inform practitioners of the tenets of evidence-based decision making, and forge strong relationships with clinicians across disciplines and geographic locations. This involvement not only helps practitioners provide the best care to their patients, it also fosters cooperation and collaboration between health sciences librarians, informationists, and the clinicians with whom they work.

## SUPPLEMENTAL FILES

**Appendix A:** ECHO Survey
