# Supplementary material for: Providing real-time resources in support of LGBTQ+ and HIV+ populations as information experts on the ECHO hub team: a case report
Source: J Med Libr Assoc. 2021 Oct 1;109(4):631–6. doi: 10.5195/jmla.2021.1262 (PMC8608201; doi:10.5195/jmla.2021.1262)
Supplement: Supplementary file 1 — Appendix A: ECHO Survey [file jmla-109-4-631-s01.docx]

Appendix A: ECHO Survey

Q1 Please rate your agreement with the following statement: as a member of the hub team, the medical librarian adds value to ECHO sessions.

- Strongly agree (1)
- Somewhat agree (2)
- Neither agree nor disagree (3)
- Somewhat disagree (4)
- Strongly disagree (5)

Q2 Before this ECHO, had you worked with a medical librarian in any professional capacity?

- Yes (1)
- No (2)
- Unsure (3)

Q3 What reason(s), if any, you would give to another hub team to include a medical librarian as a member?

Q4 What, if anything, could be improved about the way the medical librarian is currently involved in ECHO sessions?
